# Autonomous navigation of quadrupeds using coverage path planning with morphological skeleton maps

**DOI:** 10.3389/frobt.2025.1601862

**Published:** 2025-07-31

**Authors:** Alexander James Becoy, Kseniia Khomenko, Luka Peternel, Raj Thilak Rajan

**Affiliations:** ^1^ Department of Cognitive Robotics, Faculty of Mechanical Engineering, Delft University of Technology, Delft, Netherlands; ^2^ Department of Microelectronics, Faculty of Electrical Engineering, Mathematics and Computer Science, Delft University of Technology, Delft, Netherlands

**Keywords:** quadruped, autonomous navigation, unstructured environment, coverage path planning, robot operating system 2

## Abstract

This article proposes a novel method of coverage path planning for the purpose of scanning an unstructured environment autonomously. The method uses the morphological skeleton of a prior 2D navigation map via SLAM to generate a sequence of points of interest (POIs). This sequence is then ordered to create an optimal path based on the robot’s current position. To control the high-level operation, a finite state machine (FSM) is used to switch between two modes: navigating toward a POI using Nav2 and scanning the local surroundings. We validate the method in a leveled, indoor, obstacle-free, non-convex environment, evaluating time efficiency and reachability over five trials. The map reader and path planner can quickly process maps of widths and heights ranging between [196,225] pixels and [185,231] pixels in 2.52 ms and 1.7 ms , respectively. Their computation time increases with 22.0 ns/pixel and 8.17 μs/pixel, respectively. The robot managed to reach 86.5% of all waypoints across the five runs. The proposed method suffers from drift occurring in the 2D navigation map.

## 1 Introduction

Due to advancements in technology and miniaturization, surface (or ground) robots, such as wheeled and legged robots, have been increasingly adopted for diverse operations in harsh and unstructured environments in the past decade. One of the key challenges in such environments is the lack of infrastructure to support diverse operations. These environments include, for example, disaster response (Chiou et al., 2022; Lin et al., 2022; Solmaz et al., 2024), mining operations (Paredes and Fleming-Muñoz, 2021; Ai et al., 2024), space exploration (Jiang et al., 2022; Candalot et al., 2024; Arm et al., 2019; Rajan et al., 2024), surveillance in remote locations (Miller et al., 2020; Chagoya et al., 2024), or hazardous industries such as nuclear power plant maintenance (Chen et al., 2022; Sharma et al. 2024).

In such complex environments, legged robots are more versatile and robust than other surface robots, such as wheeled rovers, and they can adaptively navigate uneven, rugged, or soft terrain. Legged robots can cover relatively larger spatial areas by choosing safe footholds within their range of motion and rapidly responding to adjust their kinematic configuration (Yin et al., 2023) to achieve their objectives. The number of legs in a legged robot determines its movement efficiency and ability to maintain stability (Nitulescu et al., 2016). Compared to bipedal humanoids, quadrupedal robots demonstrate a greater load capacity and improved stability due to their broader base of support. On the other hand, quadrupeds possess simpler structures and control mechanisms than hexapodal and octopodal robots (Fan et al., 2024; Chai et al., 2022). For this reason, quadrupedal robots are ideal for tasks involving the safe navigation of complex 3D environments for (sub-) surface exploration.

**FIGURE 1:**
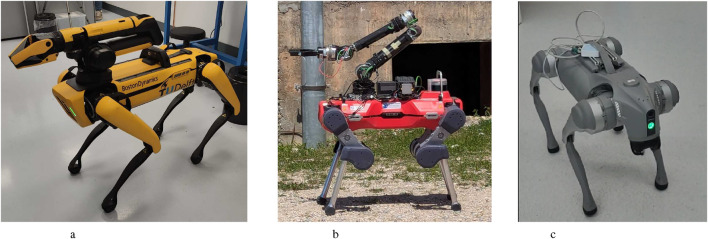
Commercially available quadrupedal robots from different companies: **(a)** Boston Dynamics Spot. **(b)** ANYbotics ANYmal D. **(c)** Unitree Robotic Go2 Edu.

Several quadrupedal robots are already commercially available in the market. We compare three notable examples, namely, Boston Dynamics’ Spot, ANYbotics’ ANYmal, and Unitree Robotics’ Go2 Edu, as shown in Figure 1, regarding attributes related to the access of development, operation durability, and affordability, as provided in Table 1. Both Spot and ANYmal have garnered significant popularity and have made substantial contributions to research and engineering (Portela et al., 2024; Zimmermann et al., 2021) compared to Go2 Edu. However, their operational runtime is limited, and their cost is considerably higher. Go2 has three modes: Air, Pro, and Edu, costing 1,600 USD, 2,800 USD, and 12,500 USD, respectively. However, only the Edu mode allows for software development, which is necessary for custom implementations, including other necessary features. Furthermore, Go2 Edu has a dedicated Robot Operating System 2 (ROS 2) integration that allows rapid development and testing. With a factor of two to three in running time, experiments can be conducted over a long session, and the robot can provide sufficient battery capacity to power additional sensors. This paper focuses on the development of software architecture for path planning and navigation specific to Go2 Edu.

**TABLE 1:** Comparison of commercially available quadrupedal robots.

Feature	Spot	ANYmal D	Go2 Edu
Manufacturer	Boston Dynamics	ANYbotics	Unitree Robotics
Dimensions (L/W/H, mm)	1,100 × 500 × 191	930 × 530 × 890	700 × 310 × 400
Maximum walking speed (m/s)	1.6	1.3	3.7
Average running time (min)	90	90–120	120–240
Integrated LiDAR	No	Yes	Yes
Integrated optical camera	Yes	Yes	Yes
Integrated depth camera	Yes	Yes	No
Connectivity	Wi-Fi, Ethernet	Wi-Fi, 4G	Wi-Fi, Bluetooth, 4G, Ethernet
Custom software development	Supported (SDK, APIs)	Supported (ROS integration, APIs)	Supported (SDK, ROS integration)
Estimated cost (1,000 USD)	74.5	150	12.5

The goal of this research is to enable quadrupedal robots to map terrain using coverage path planning. To achieve this, quadrupeds require sensors—most commonly cameras (Zhang et al., 2022; Zhang et al., 2025a; Zhang et al., 2025b) and LiDAR (Bouman et al., 2022; Niu et al., 2024). In order to scan with these sensors, the robot needs to move to various points of interest (POIs), which requires coverage path planning and navigation methods. These are examined in the related works section below.

### 1.1 Related works

Several studies have recently explored the development of software infrastructure for the Unitree Robotics’ Go2 Edu robot. Mei et al. (2024) developed a reinforcement learning method to enable the Go2 Edu robot to navigate narrow pipes using visual inputs from a depth camera. The navigation process is relatively straightforward due to the grid-like structure of the pipes, where each pipe is aligned in a straight path, with occasional protrusions serving as obstacles. Guo et al. (2024) developed a high-level path planning using a large language model, namely, OpenAI’s ChatGPT-4o, which allows the interpretation of human verbal commands and translates them into a list of executable instructions. The system integrates a depth camera with a segmentation model to effectively perceive the environment. In addition, Cheng et al. (2024) developed a motion controller for the Go2 Edu robot to traverse complex and unstructured environments using proprioceptive sensing and collision estimation only.

Autonomous navigation using quadrupedal robots is crucial for exploring complex environments; however, research on 2D coverage path planning is limited. Ly et al. (2023) achieved coverage path planning for loco-manipulation through an integrated end-to-end pipeline combining perception, optimization, and whole-body motion planning with RGB-D camera inputs. Bouman et al. (2022) presented a 2D coverage path planner for investigating unknown and unstructured environments while accounting for time-bounded and dynamic constraints and traversability risk. The advantage of these approaches is that they guarantee timely execution when the mission is time-bound, and they find a good optimal tradeoff between the maximum area coverage and the path traveled. The drawback of these approaches is that they are focused on time constraints; therefore, they do not work when the task has a variable execution time and requires time to be defined in advance. In contrast, our approach enables planning for variable times.

Some navigation methods already enable planning for variable times. Recently, Niu et al. (2024) developed a novel autonomous exploration method that uses a topological skeleton of the environment’s geometry via LiDAR, along with a finite state machine (FSM) to enable an exploratory strategy. This was demonstrated on a quadrupedal robot in an unstructured environment. These approaches have the advantage of being very adaptable in an unknown environment due to the use of a state machine that continuously checks for undiscovered areas within the map. The main difference between this work and our approach is that their method for obtaining topological skeletons uses wave propagation and Voronoi diagrams, while our method uses a morphological technique by treating the environment as an image. The impact of this is that the morphological technique simplifies the mapping and is thus computationally more efficient.

Furthermore, all the above methods of coverage path planning have specific map generation algorithms that are an integral part of the whole approach. Therefore, they are less modular, making it difficult to replace them with developing state-of-the-art mapping algorithms. In contrast, our approach can work with different types of mapping algorithms, thus making it more modular.

### 1.2 Contribution

In this work, we address the gap in the state of the art through the following key contributions.

1. We develop a control framework based on a finite state machine that switches between different operation modes to enable autonomous navigation and environmental inspection.
2. Using a prior 2D navigation map of the surroundings, the framework rapidly generates an efficient path based on the morphological skeleton of the map, which ensures coverage from small to large areas.
3. We validate the developed system through on-field experiments involving navigational tasks using the Unitree Robotics Go2 Edu robot in an indoor environment.
4. We designed an extended interface that enables the Unitree Robotics Go2 Edu to integrate with the control framework using ROS 2.
5. The whole application is open source and available at https://github.com/asil-lab/go2-autonomous-navigation

## 2 Materials and equipment

### 2.1 Hardware specifications

The Unitree Robotics Go2 Edu features three degrees of freedom (DOFs) per leg, consisting of hip, thigh, and calf hinge joints (from base to foot). It is equipped with an inertial measurement unit (IMU), an HD wide-angle camera, and foot-end force sensors. The robot offers a battery life of 2–4 h and supports fast charging^1^.

For navigation and perception, the Go2 Edu is fitted with the Unitree L1—a 4D LiDAR (3D position + 1D greyscale) based on laser time-of-flight (TOF)—mounted on its mouth. This LiDAR provides a 360° × 90° field of view (FOV), a measurement accuracy of ± 2.0 cm, and a scanning distance of up to 30 m with 90% reflectivity. It integrates an IMU with a 3-axis accelerometer and 3-axis gyroscope, has a proximal blind spot of 0.05 m, and features a sampling frequency of 43,200 points per second. Additionally, it operates with a circumferential scanning frequency of 11 Hz and a vertical scanning frequency of 180 Hz^2^. The integrated LiDAR sensor is also used for collision detection, which facilitates the differentiation between free and occupied spaces in the environment based on height. Additionally, it enables the generation of a 2D map for navigation during run-time and self-localization ability with respect to environmental features and the current state of the map.

Furthermore, the robot includes an expansion dock that houses an NVIDIA Jetson Orin, providing computing power of 40–100 TOPS. It also comes with a manual two-handed joystick controller for user operation. Additionally, in order to monitor the robot remotely, a TP-Link TL-WR802N Nano WLAN Router is added to establish a wireless connection between the robot and the operator’s computer.

Finally, we also integrated external sensors on the robot to measure ambiance characteristics such as temperature, humidity, and light intensity. The integration of these sensors is discussed in Supplementary Material A, but their usage is outside the scope of this work and is, therefore, not detailed in this paper.

### 2.2 Software specifications

The Unitree Robotics Go2 Edu robot has a dedicated software development kit (SDK), which allows custom implementations to be programmed. This SDK uses a data distribution service (DDS) as the networking middleware, which enables reliable and real-time data exchange between the program and the robot^3^.

Using DDS, a ROS application (Macenski et al., 2022) is also implemented to facilitate seamless communication between distributed robotic components, thus ensuring real-time data exchange, scalability, and interoperability across diverse hardware and software platforms.

In particular, we use ROS 2 due to its additional benefits compared to ROS 1, such as decentralization, simplicity, and user-friendliness. We use the ROS 2 Foxy on Ubuntu 20.04 to develop our proposed framework as these specifications are well-established for the Unitree Robotics Go2 Edu robot.

### 2.3 SLAM

For the robot to determine its location within the environment while simultaneously creating a map, we use simultaneous localization and mapping (SLAM). SLAM allows the robot to dynamically create a map based on the history of information and localize the robot as a function of both the current measurement and the map simultaneously (Stachniss et al., 2016). In our case, a 2D map is sufficient because the robot can only navigate on the ground surface in the x and y directions (excluding orientation in the z direction). It is worth mentioning that Go2 Edu already comes with its own SLAM implementation. However, at the time of the development, it was not readily available due to the lack of documentation. Moreover, we aim to maintain modularity so that this implementation can be applied to other quadruped platforms, with only the software modules bridging the proposed method and the robot needing adaptation. Therefore, we use Macenski’s SLAM_Toolbox to create a 2D map using a sequence of 2D laser scans as input (Macenski and Jambrecic, 2021). A 2D laser scan measures the distance to obstacles based on reflections detected by the robot’s laser scanner at specific angles and time intervals. Using a radial laser scanner, it can measure the layout of the surroundings around the robot simultaneously in one measurement time-step.

**FIGURE 2:**
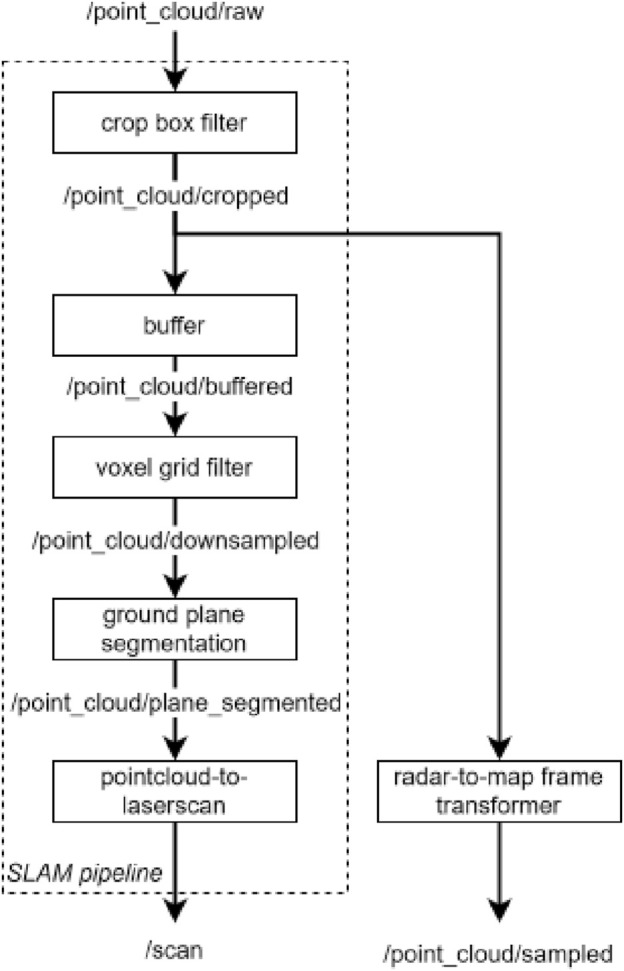
Pipeline diagram of filtering the point cloud for SLAM and 3D scanning per measurement update.

Despite the strengths of Macenski’s SLAM implementation, the integrated sensor that measures the geometry of the surroundings is a LiDAR that outputs data as a 3D point cloud. To make these data interpretable by the SLAM_Toolbox, we process the LiDAR measurements to identify the obstacles that the robot cannot navigate through. To achieve this task, we develop a data processing pipeline using the Point Cloud Library (PCL) (Rusu and Cousins, 2011), which transforms the input 3D point cloud/point _cloud/raw into the desired 2D laser scans/scan. An overview of this pipeline is shown in Figure 2. Notably, this pipeline also outputs another point cloud/point_cloud/sampled, which is later used for environment scanning, as described in Supplementary Material C.

### 2.4 Navigation

We use the Nav2 framework (Macenski et al., 2023) to ensure that the robot can plan and navigate toward a desired pose (position and orientation) in the environment, which is also completely compatible with the SLAM_Toolbox discussed previously. Using the 2D navigation map provided by SLAM, the Nav2 framework can plan and navigate a path as a function of the destination’s pose, the robot’s current pose, and the robot’s kinematic constraints with respect to its surroundings. A 2D pose is defined as $x≔{\left[\begin{array}{ccc} x & y & \psi \end{array}\right]}^{T}$ , where x and y define the longitudinal and lateral displacements, and ψ is the orientation of the robot about the z -axis (vertical displacement).

The Nav2 framework includes an array of tools such as planners, recoveries, and controllers. It is outside the scope of this paper to experiment with different tools. Therefore, we mainly used the default configuration with minor adjustments that correspond to the robot’s kinematics. Since the robot’s movement can be controlled using 2D velocities $\dot{x},\dot{y},\dot{\theta }$ as inputs, we can consider the robot to behave similarly to a differential wheeled robot. With this in mind, we can use Nav2’s default planner *NavFn Planner*.

Finally, since the Nav2 framework requires a desired pose as an input, it serves as a local planner and navigator. Therefore, in order to achieve autonomy in the robot, we require a higher-level navigation approach capable of identifying and selecting ROIs to navigate within the environment.

## 3 Methods

**FIGURE 3:**
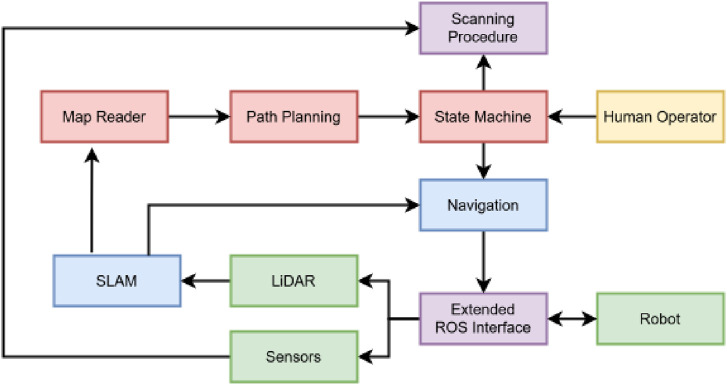
System overview of the proposed autonomous navigation with scanning capabilities in an unstructured environment. Green, hardware modules; blue, SLAM and Nav2 modules; red, autonomous navigation modules; purple, supplementary modules; and yellow, human agent.

In this work, we propose a novel framework for the Unitree Robotics Go2 Edu robot for navigation in unstructured and unpredictable environments. Figure 3 presents a system overview highlighting the key modules. The key modules that are responsible for the autonomous navigation are highlighted in red. To create a graph of ROIs, the map reader examines the SLAM-generated map and transforms it into a topological skeleton based on its geometry (Section 3.1). Path planning is informed by the graph, which creates an efficient path of ROIs, referred to as waypoints, that the robot must follow during navigation (Section 3.2). To control the high-level operation, a state machine (Section 3.3) is used to switch between actions, e.g., it checks when/whether each waypoint is complete, whether a fallback strategy is needed, and whether human operator input is detected. The communication between the modules is carried out via ROS 2 using an extended interface. More information on the extended interface can be found in Supplementary Material B.

### 3.1 Map reader

To effectively cover the environment, the robot must follow a path that ensures it traverses every corner of the space. This path should align with the trajectory of the occupied areas, e.g., corridors. This approach is generally effective under the assumption that the environment is confined within a bounded, occupied space, such as an indoor setting. In order to create the path, we process the 2D navigation map as an 8-bit array using the algorithm, as shown in Algorithm 1. M , H , and W denote the map and the map’s height and width, respectively. The map is divided into cells using the map resolution R in m/pixel. Each of these cells is an element in M , $m_{ij}$ , which only contains one of the three types of space: *occupied*, *free*, and unknown , represented by the integers 0, 255, and 128, respectively.

Algorithm 1 The 8-bit 2D navigation map M is processed into an unordered set of waypoints V, where each element is a waypoint $v\in R^{2}$ .

1: **Input:** Navigation map, i.e., $M\in N^{H\times W}$

2: **Input:** Hyperparameters $\sigma ,\kappa ,K,o\in R^{2},R$

3: V←∅

4: **for** i∈{1,…,H} **do**
5:    **for** j∈{1,…,W} **do**
6:       **if** ${\left[M\right]}_{ij}<255$ **then**
7: ${\left[M\right]}_{ij}=0$

8:       **end if**
9:    **end for**
10: **end for**
11: M← GaussianFilter( $M^{\prime }$ , σ )
12: **for** i∈{1,…,H} **do**
13:    **for** j∈{1,…,W} **do**
14:       **if** ${\left[M\right]}_{ij}>\kappa$ **then**
15: ${\left[M\right]}_{ij}=1$

16:       **end if**
17:    **end for**
18: **end for**
19: $C\leftarrow \arg\max_{\left|c_{i}\right|}$ FindContours (M)

20: M← FillAreaByContour (C)

21: M← Erode (M∣K)

22: $M_{S}\leftarrow$ Skeletonize (M)

23: **for** i∈{1,…,H} **do**
24:    **for** j∈{1,…,V} **do**
25:       **if** ${\left[M\right]}_{ij}=255$ **then**
26: $V\leftarrow V\cup \left\{R{\left[\begin{array}{cc} i & j \end{array}\right]}^{T}+o\right\}$

27:       **end if**
28:    **end for**
29: **end for**
30: **return** V

First, an indoor environment is considered. In such cases, most unknown cells inside the map are located outside the boundaries of the occupied area, e.g., beyond the walls. This is because SLAM initially represents the environment as a grid of unknown cells before any exploration occurs. Furthermore, unknown cells often persist between occupied and free regions as a result of occlusions encountered during raycasting-based measurements, such as those performed using LiDAR, as shown in Figure 6a. Consequently, this unknown space cannot be reached by the robot and can be treatedas part of the boundaries—i.e., as occupied space, as shown in Figure 6b.

To establish a smooth connection between occupied cells affected by noise in the map, a Gaussian filter (D’Haeyer, 1989) is applied using a standard deviation parameter σ∈N , as shown in Figure 6c. Subsequently, in order to restore the binary representation of the occupied and free cells, the filtered result is binarized using a threshold parameter κ∈[0,255] . A cell with a value greater than κ is classified as a free cell; otherwise, it is designated as an occupied cell.

Due to residual noise and the presence of transparent obstacles, such as windows, there may be free cells that are unreachable to the robot. To mitigate this issue, we assume that the navigable space for the robot corresponds to the largest 2D contour. First, the contours within the map are found using the marching cubes algorithm (Lorensen and Cline, 1998). The map is then reconstructed by filling the largest 2D contour with a value of 255, as shown in Figure 6d. It is worth noting that by filling only the largest contour, obstacles within that contour are not takeninto account.

To ensure that the robot maintains a safe distance from the occupied space during navigation, the free space in the map is reducedusing a morphological filter called erosion (Khosravy et al., 2017), as shown in Figure 6e. Erosion uses a structuring element K , also referred to as a kernel, which determines the width to be removed. For the sake of simplicity, we consider a square matrix of 1s as our structuring element: $K=1_{k}1_{k}^{T}$ , where k determines the length of the vector and k=1,2,3,… . The larger the k value, the larger the safe distance. In addition, the safe distance decreases as R decreases. Therefore, k∝R . This also removes narrow passages that prove impassable for the robot.

The remaining free space is reduced to a thin one-pixel-wide representation containing O pixels, which corresponds to the topological skeleton of the map’s geometry, as shown in Figure 6f. In a practical scenario, the time complexity of 2D skeletonization is mainly proportional to the total number of pixels in an image P as it iterates until the object becomes one pixel wide. Therefore, it can be considered O(P) (Zhang and Suen, 1984).

The 2D skeleton map $M_{S}$ is then flattened into an unordered set that describes the x - and y -positions of every 2D point by transforming the pixel coordinates into real-world coordinates using the origin o and resolution R of the 2D navigation map. Only those pixels for which $m_{S,ij}=255$ are considered in this transformation. Considering that the skeleton contains O pixels, it follows that there are O waypoints in V . We refer to every such 2D point defined by the skeleton as *waypoints*, which are denoted as $v_{i}\in R^{2}$, where 0≤i≤O . The waypoints serve as points of interest for the robot to visit.

### 3.2 Path planning

In order to scan the whole environment, the robot should perform the scanning procedure for each waypoint. Therefore, the objective is to determine a path that includes every waypoint the robot must visit while optimizing for time efficiency. Ultimately, this can be considered a Traveling Salesman Problem (TSP), which we approach by formulating a fast and efficient path-planning algorithm. It takes a connected acyclic graph G as the input and outputs a path P , which is the ordered set of waypoints. In other words, P is a sequence of vertices $v_{i}$ traversed by the robot.

In order to construct the graph G , we treat the unordered set of waypoints V , obtained from the map reader, as the set of vertices. Each vertex in V uniquely corresponds to the coordinate vector of a waypoint and can, for the sake of simplicity, be denoted as $v_{i}\in R^{2},\;\forall i=1\;\ldots ,O$ . Each vertex is then connected to other vertices if they are neighbors around the map’s resolution R . This should resemble the skeleton representation of the map, where the connections define the edges E of the graph G .

Algorithm 2 outlines the procedure for obtaining an efficient path $P^{*}$ for coverage exploration of the whole environment. First, there are dead ends found in the skeleton representation of the map. For the robot to cover the whole environment, we can utilize these dead ends. Each dead end is identified as a leaf vertex $v_{\text{leaf}}\in V_{\text{leaf}}$ and is typically defined with degree 1. Assuming the graph G is connected and acyclic, complete coverage of the map can be achieved by ensuring that the robot visits every leaf vertex in $V_{\text{leaf}}$ in sequence. Since all other vertices in the graph lie on the paths connecting the leaves, traversing to each leaf inherently requires passing through the intermediate vertices. As a consequence, visiting all leaf vertices implies that the entire graph has been traversed.

After identifying all leaf vertices, the leaf vertex closest to the robot’s current 2D position $x_{0}$ is selected as the initial source leaf vertex $v_{\text{leaf,start}}$ using Algorithm 3. For every source leaf vertex $v_{\text{leaf,start}}$ , we find the next nearest unvisited leaf vertex as the target $v_{\text{leaf,target}}$ using FindNearestLeaf, as defined in Algorithm 3. We find the shortest path $P^{\prime }$ between the source $v_{\text{leaf,source}}$ and the target $v_{\text{leaf,target}}$ given the graph G . To achieve this, we use the method FindShortestPath, which essentially utilizes Dijkstra’s algorithm. This algorithm has a time complexity of O(|E|+|V|log|V|) , where |V| and |E| denote the total number of vertices and the total number of edges in the graph, respectively (Barbehenn, 1998).

Algorithm 2 A fast and efficient approach to planning a path $P^{*}$ , with L vertices, using the unordered set of waypoints obtained from the map reader V , map resolution R , waypoint resolution D , and the robot’s starting 2D position $x_{0}$ .

1: **Input:** Define graph G(V,E)

2: **Hyperparameters:** R , D , $x_{0}$

3: $V_{\text{leaf,total}}\leftarrow \emptyset$

4: **for** $v_{i}\in V$ **do**
5:    **if** degree $\left(v_{i}\right)=1$ **then**
6: $V_{\text{leaf,total}}\leftarrow V_{\text{leaf,total}}\cup \left\{v_{i}\right\}$

7:     **end if**
8: **end for**
9: $V_{\text{leaf,visited}}\leftarrow \emptyset$

10: P←∅

11: $v_{\text{leaf,start}}\leftarrow \text{FindNearestLeaf}\left(x_{0},V_{\text{leaf,total}}\right)$

12: **while** $\left|V_{\text{leaf,visited}}|<|V_{\text{leaf,total}}\right|$ **do**
13: $V_{\text{leaf,visited}}\leftarrow V_{\text{leaf,visited}}\cup \left\{v_{\text{leaf,start}}\right\}$

14: $v_{\text{leaf,target}}\leftarrow \text{FindNearestLeaf}\left(v_{\text{leaf,start}},V_{\text{leaf,total}}\backslash V_{\text{leaf,visited}}\right)$

15: $P^{\prime }\leftarrow \text{FindShortestPath}\left(v_{\text{leaf,start}},v_{\text{leaf,target}}|G\right)$

16:    **for** $v_{i}^{\prime }\in P^{\prime }$ **do**
17:       **if** $v_{i}^{\prime }\notin P$ **then**
18: $P\leftarrow P\cup \left\{v_{i}^{\prime }\right\}$

19:       **end if**
20:    **end for**
21: $v_{\text{leaf,start}}\leftarrow v_{\text{leaf,target}}$

22: **end while**
23: $P^{*}\leftarrow \emptyset$

24: **for** k←0 to $\left\lfloor \frac{|P|-1}{D/R}\right\rfloor$ **do**
25: $P^{*}\leftarrow P^{*}\cup {\left[P\right]}_{\frac{D}{R}k}$

26: **end for**
27: **return** $P^{*}$

Once $P^{\prime }$ is found, each vertex $v_{i}^{\prime }\in P^{\prime }$ is appended into P . Unless some vertex $v_{i}^{\prime }$ reappears as a path of a different path $P^{\prime }$ , revisiting and scanning this position is unnecessary and should be avoided to conserve time and computational resources. Once iterated over all $v_{i}^{\prime }$ in $P^{\prime }$ , $v_{\text{leaf,start}}$ is inserted into a set of visited leaf vertices $V_{\text{leaf,visited}}$, and $v_{\text{leaf,target}}$ becomes the next source $v_{\text{leaf,start}}$ . Not only is the shortest path useful for appending $v_{i}$ into P but also for ensuring a feasible and unobstructed trajectory through occupied spaces.

This is iterated until all leaf vertices have been added and P is complete. Nevertheless, because the original distance between every two waypoints is approximately equal to the map’s resolution, a significant amountof time is spent on scanning with minimal displacement. Considering that the robot has a large scanning range capability of 30 m, which allows it to scan distances greater than the map resolution, we can increase the distance between every two waypoints to some arbitrary distance D by reducing P by a factor of D/R, where D≥R . This is achieved by selecting every D/R th element in P .

### 3.3 State machine

**FIGURE 4:**
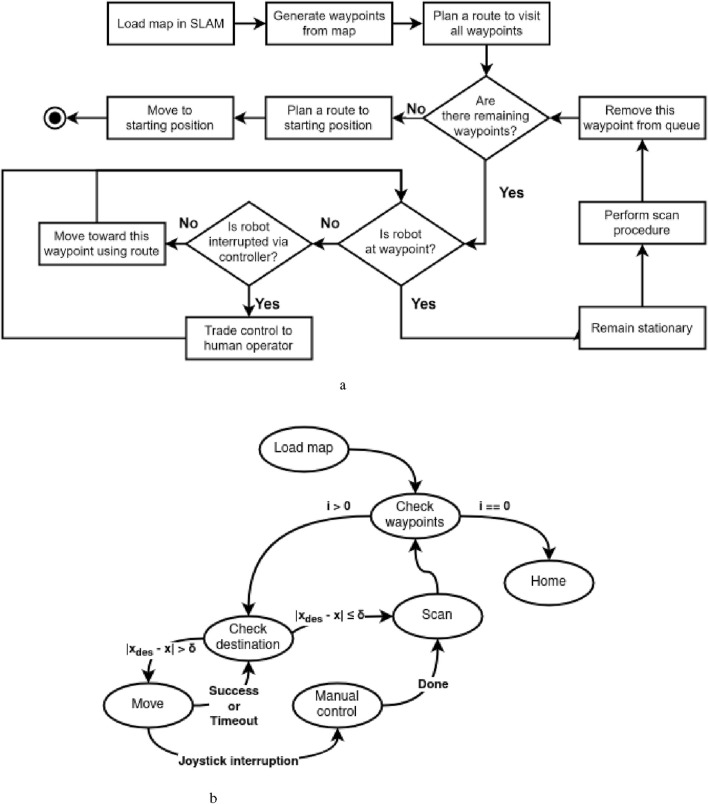
Comparison of **(a)** the activity diagram for autonomous coverage path planning and **(b)** its corresponding FSM diagram. **(a)** Activity diagram describing the autonomous coverage path planning given a prior 2D navigation map. **(b)** FSM diagram based on the activity diagram.

To achieve autonomy that enables the robot to visit the waypoints and perform a scanning procedure in succession, an activity diagram is formulated, as shown in Figure 4a. We can group two or more activities as a singular state if the transitions are consecutive without interruptions, e.g., a decision node. This state will perform all activities in their respective order. On the other hand, each decision node becomes a state that checks whether its control variable has reached a specified threshold. Each state has a conditionless trigger that automatically transitions the current state to the next specified state at the end of its action.

Algorithm 3 A method to find the nearest leaf vertex $v_{\text{leaf,target}}$ from a set of selected leaf nodes $V_{\text{leaf}}^{\prime }$ to a given source $x_{0}$ using Euclidean distances.

1: **Function** FindNearestLeaf
2: **Input:** $x_{0}\in R^{2}$ and $V_{\text{leaf}}^{\prime }$

3: $v_{\text{leaf,nearest}}\leftarrow 0$

4: d←∞

5: **for** $v_{\text{leaf,i}}\in V_{\text{leaf}}^{\prime }$ **do**
6:    **if** $|v_{\text{leaf,i}}-x_{0}|<d$ **then**
7: $v_{\text{leaf,nearest}}\leftarrow v_{\text{leaf,i}}$

8: $d\leftarrow |v_{\text{leaf,i}}-x_{0}|$

9:    **end if**
10: **end for**
11: **return** $v_{\text{leaf,nearest}}$

12: **End Function**

With a FSM, we can achieve the desired autonomous navigation based on the triggers by handling the sequence of actions. The implemented state machine diagram is shown in Figure 4b. Each state is explained as follows:

• In State: Load Map, an existing 2D navigation map is loaded into SLAM, allowing the robot to localize itselfwith respect to the map’s metadata (meter-per-pixel resolution, width and height both in pixels, and origin as (x,y) in meters) and the robot’s concurrent surroundings using 2D laser scans. After this map is loaded, it is then analyzed to generate a list of waypoints, each in (x,y) coordinates, for the robot to visit using the map reader. An optimal route is then planned to visit all of these waypoints, which reorders the original list using the path planner. Finally, it automatically transitions to the next state, State: Check Waypoints.

• State: Check Waypoints allows the system to iterate over the list of waypoints. If there are waypoints remaining, it removes the waypoint in the first entry of the list and stores it as the current destination, and it transitions to State: Check Destination. If there is no waypoint left, it transitions to State: Home.

• In State: Check Destination, the state machine determines whether the robot is already at the current destination with some acceptable offset. This is carried out by determining whether the Euclidean distance between the robot’s 2D pose x and a destination’s pose $x_{\text{des}}$ is less than or equal to a set tolerance δ , where $x,x_{\text{des}},\delta \in R^{3}$, since the poses are defined along the x - and y -axes and the yaw orientation ψ with respect to the /map frame. If the condition is true, it transitions to State: Scan. Otherwise, it transitions to State: Move. It is worth noting that the path planner does not account for the orientation ψ . However, this orientation is likely a necessary requirement of the scanning procedure, which ensures that the robot is aligned with the desired orientation, as described in Supplementary Material C.

• In State: Move, the robot is actuated to navigate toward the desired destination. Navigation is considered successful when the Euclidean distance between the robot’s current pose x and the target destination $x_{\text{des}}$ is less than or equal to a predefined threshold δ . Alternatively, if the navigation process exceeds a specified timeout duration $T_{\text{timeout}}$ , it is also terminated. In either case, the system transitions back to the Check Destination state. However, if the system detects an interruption by a human operator via the joystick controller mid-operation, the system promptly cancels the ongoing action and immediately transitions to State: Manual Control.

• In State: Scan, the robot performs the procedure to scan the local environment, which is detailed in Supplementary Material C. Once it is completed, it transitions to State: Check Waypoints.

• State: Manual Control allows the human operator to take over the robot’s navigation and move it toward the destination. This should happen in a case when the robot is traversing difficult terrains or when the navigation framework is stuck at finding the right solution. This state transitions to State: Scan once the operator presses a button on the controller.

• In State: Home, the robot travels back to its starting position. Once the robot arrives at the starting position, it lies down on the ground and waits for new commands.

To sum up, the proposed framework for navigation consists of three key modules: the map reader to extract POIs using the 2D navigation map as waypoints, the path planner to order the waypoints as an efficient route given the robot’s current position, and the state machine to enable the robot to navigate toward every waypoint and scan consecutively.

## 4 Results

To demonstrate the proposed framework and evaluate how well it performs, we conducted the experiment in a level, indoor, obstacle-free, non-convex environment. For evaluation, we used the two following metrics:

1. Time efficiency: the time required to process the map to create a path and reach each waypoint consecutively.
2. Reachability: the number of waypoints that the robot can reach over the total number of waypoints planned in %.

The Go2 Edu robot was initially manually controlled via a wireless controller to generate a 2D navigation map using the SLAM module. The experiment was then conducted, and the whole process was repeated over five trials. The SLAM module’s map resolution was set to 0.10 m. The point cloud buffer time was set to 0.25 s. The laser scan’s minimal and maximal ranges were set to 0.50 m and 30.0 m, respectively. The smoothing standard deviation was set to three. The crispification threshold was set to 128. The erosion kernel K was set to 10 x 10 pixels. The waypoint-to-waypoint distance D in path planning was set at 1.00 m. The robot’s maximum x-, y-, and yaw velocities were set to 1.00 m/s, 0.50 m/s, and 0.80 rad/s, respectively. The 2D navigation position and orientation tolerance were set to 0.05 m and 0.08 rad, respectively, with a navigation timeout of 10.0 s to replan. The scanning procedure module was disabled to demonstrate the navigational tasks.

### 4.1 SLAM, map reader, and path planning

**FIGURE 5:**
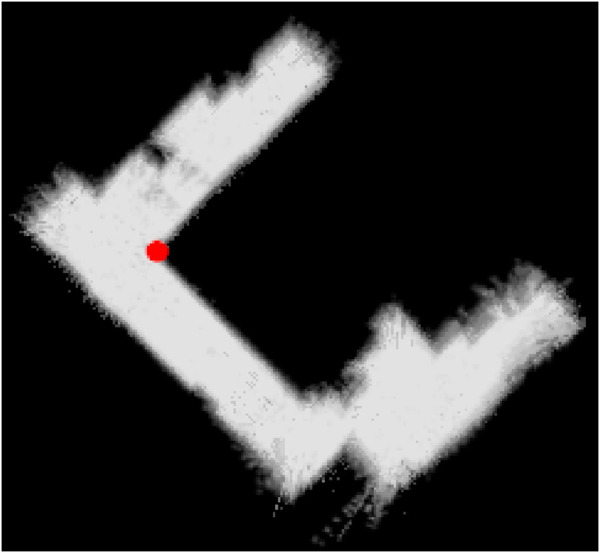
Derivation by normalizing over the five 2D navigation maps obtained from five trials, where the inner corner of the left wing of the room is taken as the origin, which is denoted as a red point. All five maps are translated and rotated around the red point accordingly.

**FIGURE 6:**
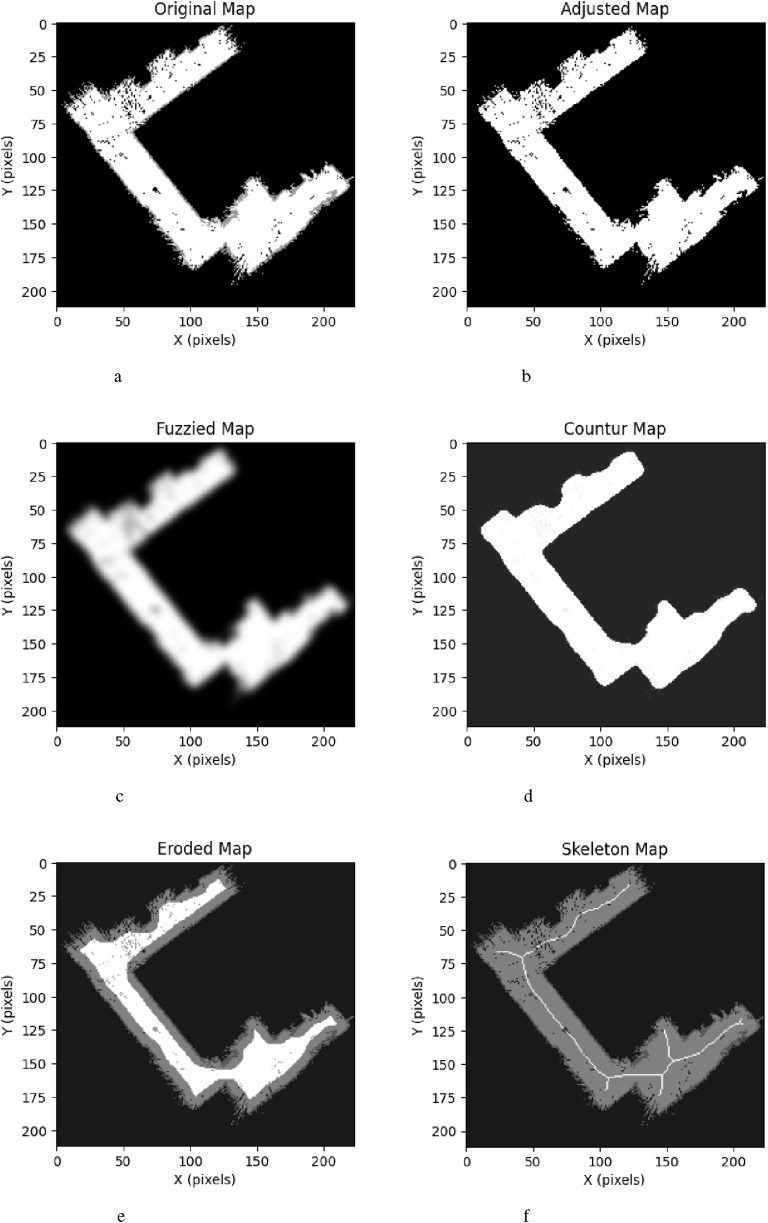
Demonstration of the map reader’s pipeline using the 2D navigation map of trial 5 as input. **(a)** Original map. **(b)** Adjusted map. **(c)** Fuzzied map. **(d)** Contour map. **(e)** Eroded map. **(f)** Skeleton map.

Before presenting the main results, we first validate the accuracy of the 2D navigation maps generated by the SLAM module. The average map is derived by normalizing over the five 2D navigation maps obtained from five trials, as illustrated in Figure 5. The width and height of these maps range between [196,225] and [185,231] pixels, respectively. We take the inner corner of the left- wing (upper left triangle of the map) of the room as the origin to align the maps in position and orientation accordingly. Analysis of the averaged map reveals that the right wing exhibits a wider range of shades of gray about the occupied space, suggesting that this part of the environment is slightly tilted relative to its actual orientation. Nonetheless, this does not severely affect the map reader, path planning, and navigation as the robot mainly localizes itself in its immediate surroundings and the topological skeleton of the map can still be found. For instance, as shown in Figure 6, the map reader can still create waypoints mainly using the map’s geometry, which also enables the robot to visit the corners by producing branches from the main path to these corners.

**FIGURE 7:**
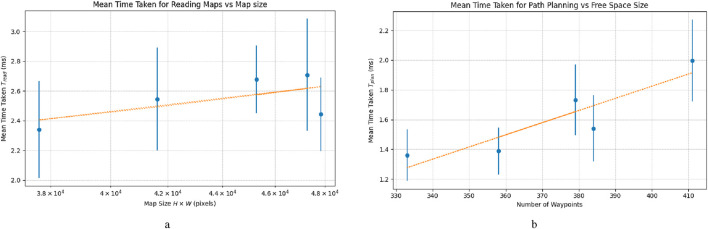
Comparison of the mean time taken for **(a)** map reader and **(b)** path planner over five trials. **(a)** Mean time taken for map reader over five trials, iterated over N = 100 plotted in blue dots, with standard deviation in 1σ as the vertical blue line. Trend line is 22.0 ns/pixel. **(b)** Mean time taken for path planner over five trials, iterated over N = 500 plotted in blue dots, with standard deviation in 1σ as the vertical blue line. Trend line is 8.17 μs/pixel.

To evaluate the time efficiency of the map reader, we record the duration required to process each map across five trials, considering a total of N=100 instances. We vary the weights in terms of the dimension per map. To facilitate a clearer representation in the plot, we use the product of the dimensions, H×W, in pixels. As shown in Figure 7a, we can observe that the average time taken for the map reader ranges from 2.34 ms to 2.70 ms over the five trials, with an average standard deviation of approximately 0.30 ms. We can also observe that the mean time increases by 22.0 ns for every additional pixel in the map. For instance, with a map of size $H\times W=10^{6}$ pixels, the expected mean time is $T_{\text{read}}\approx 23.6$ ms.

To remain within a maximum time taken of 1.0 s, the map should not be larger than the size of $H\times W=45.4\times 10^{6}$ pixels. Assuming a square map with a map resolution of 0.10 m, this maximum map has a length of 673.4 m. Hence, our proposed map reader is fast for relatively small to large map sizes (ranging up to $45.4\times 10^{6}$ pixels) within a lead time of $T_{\text{read}}=1.0$ s, especially when the map reader is only run once to produce the waypoints given the map’s geometry.

To determine the path planner’s time efficiency, we repeat the same process with the evaluation of the map reader over N=500 iterations per trial. Instead, we vary the weights in terms of the number of waypoints per map. In Figure 7b, we can observe that the mean time taken ranges between 1.40 ms and 2.00 ms. The variances can be explained by the additional time required to find the next nearest leaf vertex from a source vertex as it searches the list sequentially. According to the trend line, the lead time increases by 8.17 μs for every additional waypoint. Therefore, according to the trend line, it means that for a map with 1,000 waypoints, the planning time becomes $T_{\text{plan}}\approx 6.18$ ms, and for a map of $10^{6}$ waypoints, the planning time is $T_{\text{plan}}\approx 8.17$ s.

To stay within a lead time of 1.0 s, the number of waypoints should not be larger than $1.219\times 10^{5}$ . The bottleneck appears if the map resolution R becomes small as it increases the number of pixels per meter in a map. This can substantially increase the number of waypoints by a factor of $R/R^{\prime }$ , where $R^{\prime }$ is the new map resolution and $R^{\prime }<R$ . Nonetheless, the path planner can be considered fast for a small to large number of waypoints, given a reasonable map resolution R . It is also worth noting that it also runs only once to produce the necessary path.

**FIGURE 8:**
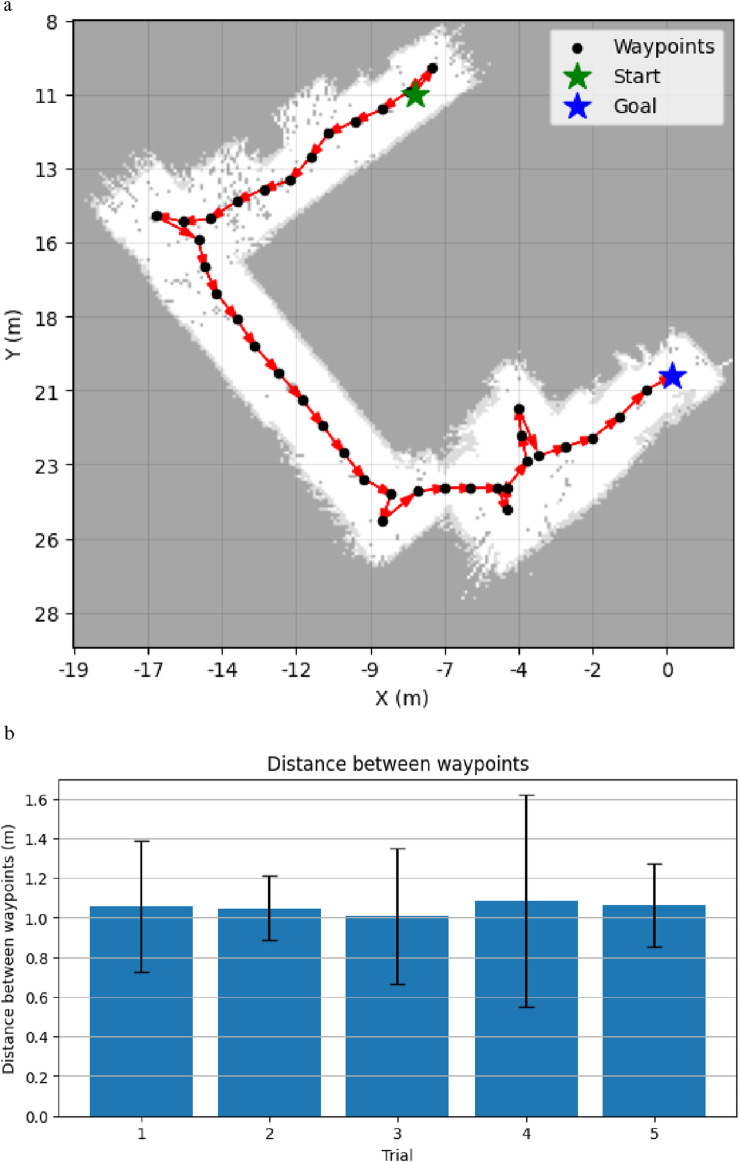
Demonstrations of path planner’s performance. **(a)** An efficient path to explore the whole environment from the robot’s starting position (green) to the farthest waypoint (blue) while visiting all other waypoints (black). Each arrow (red) denotes the direction from the source to the target. **(b)** Mean distance between every two connected waypoints generated by the path planning per trial, as defined by the waypoint resolution D.

To verify the path planner’s performance, we first evaluate the logic of whether it works as intended. Using one trial as an example, we observe that the path planner has created an optimal path in terms of time, based on the given waypoints and the robot’s current 2D position, as shown in Figure 8a. Furthermore, according to Figure 8b, we can observe that the mean waypoint–waypoint distance is approximately 1.00 m, given the previously set waypoint resolution D . The variance can be explained by the fact that the robot skips waypoints that have already been visited as it is unnecessary to scan the same position twice. This results in the robot having to travel distances of 1.00 m or more for some intervals. In contrast to traveling a longer distance, the robot can also visit the next waypoint in a shorter distance. This is because the path splicing happens at the end of the algorithm, whereby it results in a probability that two waypoints are less than the set waypoint resolution. Nevertheless, this does not heavily affect navigation, and the average waypoint distance is almost identical to the set waypoint resolution D .

### 4.2 Navigational performance

**FIGURE 9:**
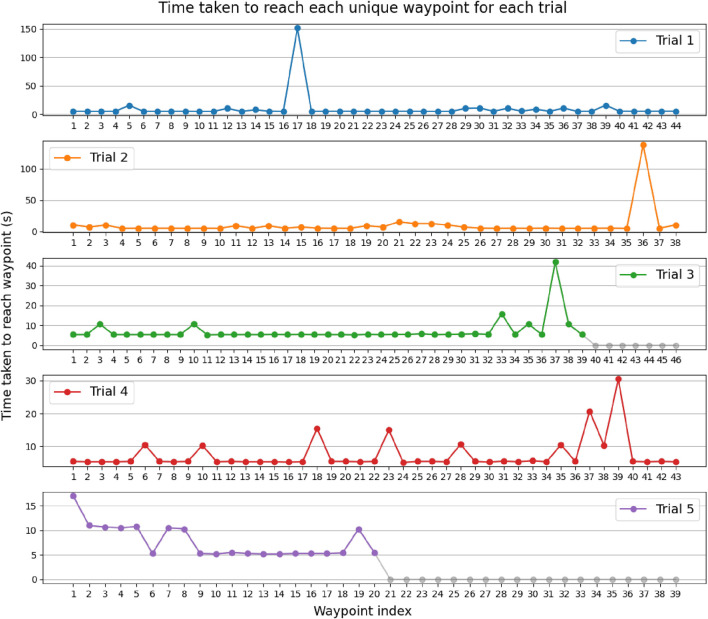
Time taken in seconds for the robot to reach each possible unique waypoint over five trials. Gray dots describe waypoints that the robot has not been able to reach. The outliers occurred due to Nav2 getting stuck at replanning, such that the navigation terminates within narrow desired tolerances, or due to map drift.

To determine the robot’s navigational performance, we evaluate it according to the two aforementioned metrics, as shown in Table 2. In addition, Figure 9 shows the time taken for each waypoint over all trials. We can observe that the robot is able to reach 86.5% of the total waypoints across all trials, with a median time of 5.38 s per waypoint. As shown in Figure 9, this was achieved in an average time of 8.525 s, with a standard deviation of 11.4 s.

**TABLE 2:** Reachability and time taken for each trial, including key observations.

Trial	Reachability (%)	Total time (s)	Median time per waypoint (s)	Observations
1	100.0	443.2	5.40	Human assistance required at waypoint 17
2	100.0	397.0	5.30	Human assistance required at waypoint 36
3	82.61	287.2	5.40	Significant map drift occurred after 3 min, such that the remaining eight waypoints were unreachable (displaced into the occupied space)
4	100.0	322.3	5.40	Robot stalled at waypoints 6, 10, 18, 23, 28, 35, and 37–39 due to replanning
5	48.72	160.1	5.40	Significant map drift occurred after 2 min, such that the remaining 19 waypoints were unreachable as they displaced into the occupied space

We can see several outliers where the robot took time ranging from 10.0 s to a few minutes to complete the navigation to the adjacent waypoint, for instance, at waypoint 17 in trial 1. This can be explained by Nav2 trying to create a local path that ends precisely within the desired tolerances in position and orientation, and because the robot requires a minimal input velocity in order to move, it overshoots the target, causing Nav2 to replan the local path. This can result in the robot becoming stuck indefinitely and requires assistance from a human operator. For some of these outliers, ranging from 30 s, human assistance is eventually required to reposition the robot correctly within the desired position and orientation tolerance.

**FIGURE 10:**
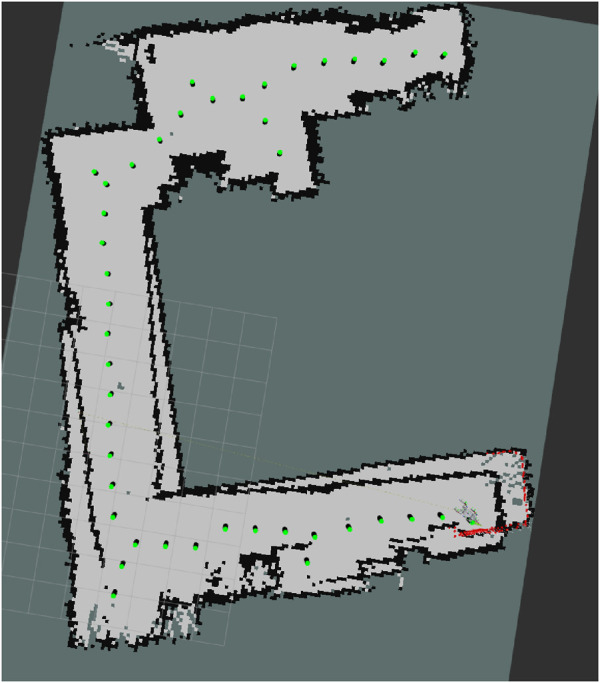
Drift in the map where the hall is doubled. The drift occurred 10 min after the autonomous navigation started in trial 3.

Furthermore, Table 2 shows that in trials 3 and 5, the robot failed to reach all of the waypoints. This is mainly due to map drifts that occur over time and the fact that the SLAM module was still operating by mapping the robot’s surroundings online. This can be recognized, e.g., as two identical hallways slightly tilted relative to one another, as shown in Figure 10. Due to the drift in the navigation map, since the coordinates of the waypoints remain the same, SLAM adjusts the 2D navigation map such that the later waypoints appear in places that the robot cannot reach, such as in the walls. Drift usually occurs because we only use relative sensors to localize itself with respect to the surroundings, e.g., IMU, leg encoders, and LiDAR. This can cause the uncertainty to increase over time, which is inherent in odometry.

## 5 Discussion

Coverage path planning allows mobile robots such as quadrupeds to explore the whole environment. This is especially useful for applications such as surveillance, inspection, and search and rescue. Nevertheless, limited work has been carried out on autonomous navigation using coverage path planning on quadrupeds. Therefore, we developed an open-source framework using ROS 2 that enables a Unitree Robotics Go2 quadruped to autonomously navigate and visit every corner using a prior 2D navigation map. It utilizes a map reader to extract a graph of 2D waypoints using the topological skeleton of the map and a path planner to create an efficient path with respect to time and the starting position. A state machine is used to iterate over the ordered list of waypoints and navigate them in succession.

The map reader and the path planner can quickly process maps with widths and heights ranging from 196,225 pixels to 185,231 pixels in 2.52 ms and 1.7 ms, respectively. Their computation times increase with 22.0 ns/pixel and 8.17 μs/pixel, respectively. In a closed and unstructured environment, the robot managed to reach 86.5% of all waypoints over five runs. The failure can be explained due to drifts occurring in the maps over time because SLAM still operates online. Map drifts can be mitigated using absolute sensors such as global positioning system (GPS) and ultra-wideband anchors. Another issue that our path planning does not take into account is obstacles inside the large free space. This can be mitigated by subtracting the contours of said obstacles from the large free space. Skeletonization will account for the creation of waypoints around these occupied spaces. However, the presence of obstacles may necessitate adjustment to the path planner as they can give rise to cyclical graphs.

Compared to the state-of-the-art methods that use time-constrained planning (Bouman et al., 2022; Ly et al., 2023) and do not enable variable task execution times, our approach is not constrained by a predefined time. While a time-constrained approach is useful when the mission time is predefined, the variable-time approach provides a more adaptable solution when operating with limited knowledge. Unlike alternative state-of-the-art approaches that enable time-variable exploration (Niu et al., 2024), our approach is more computationally efficient due to the use of a morphological technique to extract the topological skeleton of the map. Even though the experimental conditions were different, a rough comparison of the map generation magnitude based on the results from each paper shows an order-of-magnitude difference (milliseconds vs. microseconds).

Nevertheless, the proposed method is primarily suited for known and static environments, rendering it unsuitable for real-world applications with irregular and moving obstacles within the environment and varying elevations. Future work will, therefore, focus on extending the proposed method to incorporate real-time autonomous coverage exploration in unknown 3D environments, particularly under diverse environmental conditions and in the presence of irregular and dynamic obstacles. Additionally, the influence of variations in key parameters will be systematically investigated. Moreover, the study will also investigate and aim to improve drift issues commonly encountered in SLAM. Finally, the proposed approach will be systematically compared with existing 2D coverage path planning methods to evaluate its relative performance and advantages.

## Data Availability

The raw data supporting the conclusions of this article will be made available by the authors, without undue reservation.

## Footnotes

1 Go2 SDK Development Guide - About Go2 https://support.unitree.com/home/en/developer/about_Go2

2 4D Lidar L1 Application Scenarios 4D Lidar L1 Efficacy — Unitree Robotic https://www.unitree.com/LiDAR

3 Go2 SDK Development Guide - SDK Concepts https://support.unitree.com/home/en/developer/SDK_Concepts
