# Supplementary material for: Autonomous navigation of quadrupeds using coverage path planning with morphological skeleton maps
Source: Front Robot AI. 2025 Jul 31;12:1601862. doi: 10.3389/frobt.2025.1601862 (PMC12351651; doi:10.3389/frobt.2025.1601862)
Supplement: Supplementary file 1 [file DataSheet1.pdf]

# Supplementary Material: Autonomous Navigation of Quadrupeds Using Coverage Path Planning with Morphological Skeleton Maps

## 1 SENSING AMBIANCE

Sensing additional parameters of the environment is important for improved awareness, as well as for the robot's usability in various exploration use cases. The robot is equipped with two additional sensors: a sensor `Adafruit BH1750` that measures the light intensity (in lux), and a sensor that measures both the temperature (in C) and the humidity (in %) `Adafruit SHT31`. These sensors are connected to an `Arduino 33 BLE` micro-controller via the I2C interface. This microcontroller is then connected to the robot via the USB interface. This is illustrated in Fig. S1.

When the system starts up, it initializes the sensors and begins a serial port with the robot with the baud rate of 115200 Bd. Afterwards, it periodically[1] stimulates the sensors to measure at an instantaneous time. These measurements are quantized as 32-bit floating points. These floats are then transferred to the robot via the serial port with their bit-wise representations. It is important to note that the order is similar between the robot and the microcontroller. This implementation can be seen in `auxiliary_sensing` in the root directory.

The ROS 2 node `auxiliary_sensors_node` from the package `ltm_go2_auxiliary_sensors` creates a CSV-format file and it also connects to the Arduino's serial port upon startup, and translates every incoming packet into its 32-bit floating point representations. This is then published onto the topic `/ambient_state`. This information is also added as a line into the CSV-format file with the corresponding timestamp.

## 2 EXTENDED ROS INTERFACE

ROS has existing tools and libraries that roboticists can swiftly plug and play into their workspace. One of these libraries is a visualization program called RViz that allows us to see what the robot is doing and perceiving in 3D space. The data are only available in Unitree's custom ROS message types, whereas RViz uses common ROS message types mentioned in [2].

Another library is TF2, which allows us to keep track of multiple reference frames, maintain tree-structured relationships between them using the kinematic model of the robot, and transform points, vectors, etc. from one reference frame to another at any desired moment in time. This is convenient when we want to observe the robot in an inertial reference frame that is not moving along with it. TF2 uses the message type `geometry_msgs/TransformStamped` to regularly update the transform between the child reference frame and its parent reference frame.

Besides visualization and transformation, we need to be able to control the robot to perform tasks such as navigation, as well as perform gestures such as sitting down. To fully draw on the available raw data

[1] The measurement rate is arbitrary, here we use $f = 5000$ Hz. The minimum frequency should be equal to the sensor that has the highest throttle time.

[2] Strictly speaking, it is possible to visualize custom message types in RViz if you implement custom RViz plugins. However, this requires additional time and effort.

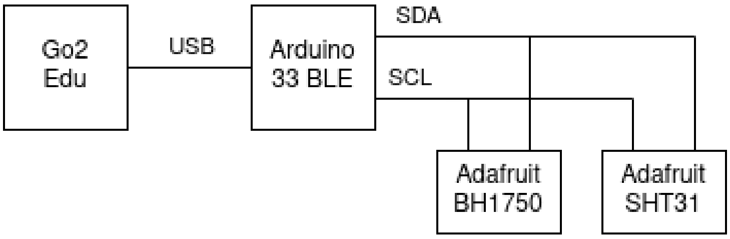


Figure S1: A block diagram illustrating the interconnections between the robot Unitree Robotics Go2 Edu, the microcontroller Arduino 33 BLE, and the sensors Adafruit BH1750 and Adafruit SHT31.

and services, we need an interface to format the data into the corresponding common message types, request services to perform certain tasks, and determine which data we should utilize for visualization and scanning purposes. In addition, to visualize the model of the robot, we use the available URDF of Go2 from `unitree_ros` with minor modifications. The following sections describe the interfaces each processing different data types.

## 2.1 Odometry

Unitree Go2 has a built-in odometry that defines the robot transformation from some inertial reference frame named `odom` to the robot's body reference frame named `base_link`. Let us denote these as $\mathcal{N}$ and $\mathcal{B}$, respectively, and we denote the transformation as ${}^{\mathcal{B}}C_{\mathcal{N}}$[3]. This inertial reference frame is defined as the 3D position where it is initially booted up.

The odometry data is stored in the `unitree_go/SportModeState` message on the topic `/sportmodestate`. To update the transformation ${}^{\mathcal{B}}C_{\mathcal{N}}$, we use position for translation, and quaternion in the message's `imu_state` for rotation.

Furthermore, it is also worth publishing the IMU data as a separate entity to record the history. This data is published on the topic `/imu` using `sensor_msgs/Imu` message type. This contains the IMU's measured orientation as `geometry_msgs/Quaternion`, angular velocity as `geometry_msgs/Vector3`, and linear acceleration as `geometry_msgs/Vector3`.

Besides this, another frame is created that is projected from the robot's `base_link` frame normal to the ground called `base_footprint` $\mathcal{F}$. This is used to project the 2D navigation map onto $z = 0$ when $\mathcal{N}$ as well as the obstacles in the point cloud, as $\mathcal{B}$ is defined at the robot's height. To obtain for the rotation between `base_link` and `base_footprint` frames ${}^{\mathcal{F}}C_{\mathcal{B}}$, we expand the rotation ${}^{\mathcal{F}}C_{\mathcal{N}}$:

$$ {}^{\mathcal{F}}C_{\mathcal{N}} = {}^{\mathcal{F}}C_{\mathcal{B}}{}^{\mathcal{B}}C_{\mathcal{N}} \Rightarrow {}^{\mathcal{F}}C_{\mathcal{B}} = {}^{\mathcal{F}}C_{\mathcal{N}}{}^{\mathcal{B}}C_{\mathcal{N}}^{-1} \tag{S1} $$

Since `base_footprint` is merely a projection of the `base_link` onto the ground plane in `odom` frame, this means that no rotation is applied. Hence ${}^{\mathcal{F}}C_{\mathcal{N}}$ is simply an identity matrix $I$. Hence:

$$ {}^{\mathcal{F}}C_{\mathcal{B}} = {}^{\mathcal{B}}C_{\mathcal{N}}^{-1} \tag{S2} $$

[3] Orientations and rotations are defined in quaternions to avoid the singularity. But, for the sake of clarity, we will be using rotation matrices instead.

The translation $x, y, z$ in $\mathcal{F}$ depends on the height displacement $z$ in $\mathcal{B}$, because it is only a vertical projection onto the ground plane. Therefore the translation vector of $\mathcal{F}$ with respect to $\mathcal{B}$, $\mathbf{x}_{\mathcal{B}}^{F}$, is defined as follows:

$$\mathbf{x}_{\mathcal{B}}^{\mathcal{F}} = \begin{pmatrix} x_{\mathcal{B}}^{\mathcal{F}} \\ y_{\mathcal{B}}^{\mathcal{F}} \\ z_{\mathcal{B}}^{\mathcal{F}} \end{pmatrix} = {}^{\mathcal{F}}C_{B} \begin{pmatrix} 0 \\ 0 \\ -z_{\mathcal{N}}^{\mathcal{B}} \end{pmatrix} = {}^{\mathcal{B}}C_{N}^{-1} \begin{pmatrix} 0 \\ 0 \\ -z_{\mathcal{N}}^{\mathcal{B}} \end{pmatrix} \tag{S3}$$

## 2.2 Joint state

To visualize the current configuration of the robot on RViz, as well as to update every reference frame on the robot, for instance, when the robot is walking each joint rotates periodically, we would need to extract the angle (joint position) from each of these joints and insert it into a message type specifically designed to track the state of every joint for every measurement. This message type is called `sensor_msgs/JointState` which consists of joint names, joint positions, joint velocities, and joint efforts. Note that these are properly ordered, e.g. the first element from each of these lists corresponds to the same joint.

The robot has four legs: front right (FR), front left (FL), rear right (RR), and rear left (RL). Each leg has three joints for every part: hip, thigh, and calf. These joints rotate around the axes $X$, $Y$, $Y$ with respect to the base_link reference frame (the reference frame placed directly on the pivot center of the robot), respectively.

## 2.3 Point cloud

The point clouds are previously published as a `sensor_msgs/PointCloud2` message on the topic named `/utlidar/pointcloud` by Unitree. This is measured with respect to the radar frame.

However, the message's header is not filled, which will cause an issue when transforming the point cloud from the radar frame to a desired frame. Therefore, an interface for the point cloud must be created.

This interface acts simply as a passthrough filter while filling the received PointCloud2 message's header with the correct data:

- Frame ID: Set as 'radar' to correspond to the correct link name in the URDF.
- Timestamp: Unfortunately, we do not have access to the exact time the point cloud was created. So, we set it to the current time it was received.

This PointCloud2 message is then published on the topic `/point_cloud/raw`.

Additionally, we would also like to request the last received point cloud at any time. This is especially useful for the scanning procedure. Therefore, a service is created named `get_pointcloud` which returns the last published point cloud.

## 2.4 Control

The robot is usually controlled manually with a wireless controller. The controller is velocity-based, meaning that depending on the displacement of a joystick, the velocity changes accordingly. The output of this controller can be read on the topic `/wirelesscontroller`.

The robot can also be controlled by publishing messages into this topic with the corresponding message type `unitree_go/WirelessController`. It contains four real variables `lx`, `ly`, `rx`, and `ry`[4]. `lx` and `ly` define the displacement of the left joystick from its normal position (center), and `rx` and `ry` likewise with the right joystick. The following further describes these variables:

- `lx`: Translate the robot laterally, where positive moves the robot to the right, and negative to the left. Normalized in range $(-1, 1)$.
- `ly`: Translate the robot longitudinally, where positive moves the robot forward, and negative backward. Normalized in range $(-1, 1)$.
- `rx`: Rotates the robot about its yaw, where positive rotates the robot clockwise, and negative counter-clockwise. Normalize in range $(-1, 1)$.
- `ry`: Rotates the robot about its pitch, where positive tilts the robot to the ground, and negative to the sky. Normalize in range $(-1, 1)$.

The output of the navigation framework is the desired velocity that the robot must follow in order to traverse the planned trajectory. This output is in `geometry_msgs/Twist`, which is a message that contains the linear and angular velocities, $\mathbf{v}_x$ and $\mathbf{v}_\theta$, respectively. We can map the desired velocity into the controller.

$$l_y = v_{x,x} \tag{S4}$$

$$l_x = -v_{x,y} \tag{S5}$$

$$r_x = -v_{\theta,z} \tag{S6}$$

Note that $\mathbf{v}_x, \mathbf{v}_\theta$ follow the convention that $x$ always points forward, and the right-hand rule. Furthermore, `ry` is left untouched as it is not necessary to tilt during navigation, and the fact that the output is only defined on a 2D space.

## 2.5 Gesture

Besides the periodic movements such as walking, the robot can also perform customized episodic movements such as standing up, dancing, lying down, etc. We name these types of movements as *gestures*. Besides using the wireless controller, these gestures can only be used behind an API. Therefore, one must request the API in order for the robot to perform the gesture. This can be done by publishing a `unitree_api/Request` message with the correct API ID.

Since the LiDAR is pointing towards the ground, we would like the robot to be able to tilt it more towards the sky to achieve a scan of a larger portion of the region of interest. Tilting the robot using `ry` variable is not sufficient as it only displaces five degrees at most. The robot can perform the gesture to *sit down*, where it tilts the LiDAR farther up. Besides this, we should also be able to make the robot to *stand up* from sitting down, as well as to *lie down* before shutting down or to conserve energy.

Upon further investigation, the robot has two different modes of standing: balanced, and fixed. Balanced standing allows the robot to balance itself by dynamically moving its joints such that its center of mass

[4] It also contains an unsigned integer variable named keys which outputs each button as a binary bit and has a corresponding significant position within the variable. '1' is output when pressed, '0' otherwise. However, it is shown that this variable does not do anything using messages.

**Table S1.** A list of gestures that are implemented in the extended ROS interface.

| Gesture | Code | API ID |
|---|---|---|
| Fixed standing | `stand_up` | 1004 |
| Lie down | `stand_down` | 1005 |
| Balanced standing | `recovery` | 1006 |
| Sit down | `sit` | 1009 |

remains in the margin of stability. Whereas fixed standing ensures that all joints are fixed to specific joint positions such that they appear to be standing still. The former can be achieved using the *Recovery* gesture[5], and the latter with the *Standing*.

An interface is implemented to call the gestures with ease using a string as a code. This is done by publishing the code into a `std_msgs/String` message on the topic `/gesture`. Table S1 lists the gestures that are implemented in the interface with their corresponding code in string, and API ID:

## 2.6 Camera

The robot is equipped with a 2D camera at the front. It is accessible using OpenCV's video capture method. Note that the camera address and the port must be set correctly, as well as the multi-cast interface address. The latter depends on which hardware the interface is running. If the interface is running locally on the robot, `eth0` is inserted. If it is running on an external computer via Wi-Fi, then `wlp2s0` is inserted.

Using `cv_bridge`, we can quickly format each captured frame by OpenCV into a `sensor_msgs/Image` message. This interface is continuously publishing a sequence of images on the topic `/camera/raw` at a rate of 24 Hz, however, it is configurable and depends on the hardware's limitation. In addition to this, the most recent image can also be requested by calling the service `/get_image`. This is especially useful for the scanning procedure.

# 3 SCANNING PROCEDURE

The scanning procedure is performed for every waypoint. We are interested in scanning the environment from every angle at a given location as much as possible. However, this proves to be difficult when the robot can only scan a huge part of the environment when it is sitting. Since sitting down is a discrete motion, this means that it will take a long time when we are scanning for every small angular displacement. It is also worth noting that the robot can only work for several hours as it is running on a battery and depending on the effort. Therefore, we discretize the scanning procedure based on the number of orientations, and the desired gestures.

Algorithm 1 displays the implemented algorithm to perform the scanning procedure. It takes two inputs: $N$ is the number of orientations, and $G$ is a list of gestures to be performed, e.g., standing up and sitting down. In short, it captures the 3D point cloud and the 2D image in the exact pose for every gesture for each orientation.

[5] The Recovery gesture also allows the robot to recover from a fall.

**Algorithm 1** The pseudocode to perform the scanning procedure at a waypoint.

**Input:** $N \geq 1,\ |G| \geq 1$<br>
Get current robot's 2D pose $x, y, \theta$<br>
$n \leftarrow 0$<br>
**while** $n \neq N$ **do**<br>
    **for** $g$ in $G$ **do**<br>
        Perform gesture $g$<br>
        Capture 3D point cloud<br>
        Capture 2D image<br>
    **end for**<br>
    $\theta \leftarrow \theta + 2\pi/N$<br>
    Navigate towards 2D pose $(x, y, \theta)$<br>
    $n \leftarrow n + 1$<br>
**end while**
